# Human *Babesia microti* Incidence and *Ixodes scapularis* Distribution, Rhode Island, 1998–2004

**DOI:** 10.3201/eid1304.061035

**Published:** 2007-04

**Authors:** Sarah E. Rodgers, Thomas N. Mather

**Affiliations:** * University of Rhode Island, Kingston, Rhode Island, USA; 1 Current affiliation: Swansea University, Wales, United Kingdom

**Keywords:** Incidence studies, area analysis, babesiosis, *Ixodes scapularis*, Lyme disease, *Borrelia burgdorferi*, dispatch

## Abstract

Distribution of nymphal *Ixodes scapularis* in Rhode Island was used as a logistical regressor for predicting presence of human babesiosis. Although the incidence of babesiosis is increasing in southern Rhode Island, large areas of the state are free of babesiosis risk.

In recent years, cases of human babesiosis have increased across the northeastern United States, especially in coastal areas like southern Rhode Island. In the northeastern United States, human babesiosis is a tick-transmitted, malarialike infection caused by *Babesia microti* Franca parasites (*1*). The *B. microti* parasite shares the same principal rodent reservoir (white-footed mouse, *Peromyscus leucopus*) and tick vector (*Ixodes scapularis*) as the Lyme disease spirochete, *Borrelia burgdorferi*. Although the transmission processes of *B. burgdorferi* and *B. microti* are similar, *B. burgdorferi* is acquired nearly twice as often as *B. microti* (*2*). This less efficient transmission of *B. microti* seemingly requires more tick bites to maintain similar zoonotic prevalence. In a previous study, we suggested that a threshold of >20 nymphal ticks collected per hour was necessary to maintain zoonotic endemicity of *B. microti* in white-footed mouse populations (*3*). In this study, we used *I. scapularis* abundance to indicate the spatial distribution of risk for human babesiosis. We focused on babesiosis transmitted by ticks, not other sources such as blood transfusions (*4*).

## The Study

**Figure 1:**
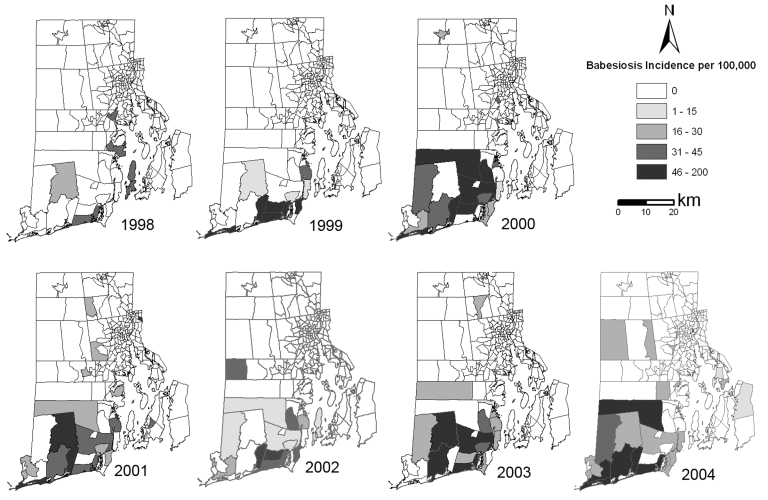
Human babesiosis incidence per census tract, Rhode Island, USA, 1998–2004. Data from Rhode Island Department of Health

Human babesiosis is not a nationally reportable disease; however, in Rhode Island all clinically diagnosed cases have been recorded by the Rhode Island Department of Health since 1994, when 2 cases were reported. A decade later, in 2004, 48 cases were reported. We analyzed all cases reported to the Rhode Island Department of Health from 1998 through 2004; the number of cases before 1998 was insufficient for analysis. Of 189 babesiosis patients, mean age was 59 years (SD 20.42), and 57% were >60 years of age. The case rate in Rhode Island more than doubled each year from 1998 through 2000, after which the rate of increase slowed. Geographic (latitude, longitude) coordinates of each patient’s home address were determined by using EZlocate (www.geocode.com). Cases were aggregated into Rhode Island’s 233 census tracts by using ArcMap (Environmental Systems Research Institute, Inc., Redlands, CA, USA). Spatial distribution of babesiosis incidence per 100,000 persons per year is shown in Figure 1. Most tracts, particularly those in urban areas (smaller census tracts), contain no babesiosis cases.

An extensive surveillance program for *I. scapularis* nymphal ticks has been in operation continuously since 1993. According to a random stratified design, sampling has occurred at least at the same 60 locations throughout Rhode Island (*5*). Forested areas, suitable as tick habitats, were drag-sampled twice each year; results were recorded as nymphs collected per hour. *I. scapularis* samples were taken each year from late May through the end of July, a time of peak nymphal activity; more detail on the sampling method can be found elsewhere (*5*,*6*). This type of surveillance is labor intensive; however, we did not routinely test ticks or rodents for *B. microti* infection. Rates for nymphs collected per hour were first interpolated by ordinary kriging (Gaussianprocess regression) to create a continuous tick-encounter surface. Variograms (to show spatial correlation of observations) were calculated from each year’s tick data and used to provide data for kriging (*7*). Subsequently, this surface was averaged for each census tract area.

**Figure 2:**
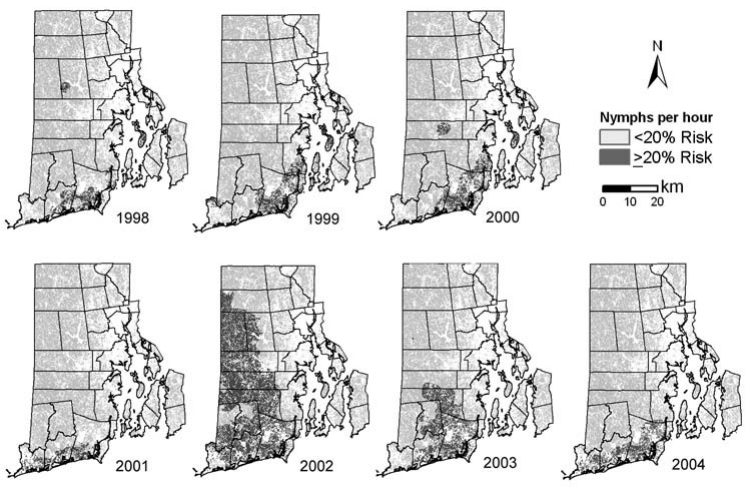
Risk for human case of babesiosis, Rhode Island, USA, derived from simple logistic regression analysis of census tracts with and without babesiosis cases (Figure 1), predicted by average *Ixodes scapularis* nymphs collected per hour per census tract. The cut-off level for the 2 classes was decided by the number of nymphs collected per hour needed to create a 20% probability of a babesiosis case occurring in a census tract. The continuous nymphal tick abundance surface was modified to subtract areas unsuitable for tick habitat because they contain water, unforested agriculture, or urban land.

Associated with each census tract are nymphal tick abundance (nymphs collected per hour) and disease presence or absence. Simple logistic regression was performed by using SAS (SAS Institute, Cary, NC, USA). Nymphal tick abundance was used as the predictor for the presence of disease; regression parameters are given in the Table. Each logistic regression curve (not shown) allowed us to ascertain an acceptable threshold of risk for nymphal tick collection per hour, below which it was unlikely (<20% probability) that a census tract would contain any cases of human babesiosis for that year. Risk is a continuous measure, and this cut-off represents the authors’ value judgment. The minimum threshold required to cause ≥20% risk of having 1 babesiosis case in any particular census tract was, for example, 135 nymphs per hour in 1998 but only 19 nymphs per hour in 2004. Nymphal threshold figures were used to create maps from the continuous nymphs-per-hour data (Figure 2). These effectively classified Rhode Island into 2 distinct zones; 1 that appears to be safe from risk for tick-transmitted human babesiosis and 1 where residents and visitors are at risk. In general, the trend was a lowering of the nymphal tick abundance threshold associated with babesiosis over the course of the 7-year study.

**Table:** Coefficients of simple logistic regression of *Ixodes scapularis* nymphs collected per hour, Rhode Island, USA

Year	Intercept	Slope	No. nymphs/h*
1998	−4.610	0.024	135
1999	−5.671	0.078	55
2000	−3.474	0.040	52
2001	−3.859	0.024	102
2002	−4.106	0.093	30
2003	−3.529	0.079	27
2004	−2.566	0.064	19

*Minimum no. nymphs that must be collected per hour to create a medium-high risk for babesiosis (20% probability of ≥1 case of human babesiosis per census tract).

## Conclusions

These results support earlier research findings that *B. microti* infections in mice are closely linked to abundance of nymphal ticks; a threshold of ≥ 20 nymphs per hour is most likely to support enzootic parasite maintenance and potentially result in human disease (*3*). Clearly, areas of highest risk for babesiosis transmission are in southern Rhode Island, where the highest incidence of nymphal ticks and zoonotic babesiosis infections in rodent reservoirs are located. However, we observed a marked increase in human babesiosis incidence in Rhode Island from 1998 through 2004. This increase likely results in part from the expanding area in Rhode Island where numbers of nymphal ticks reach or exceed the proposed zoonotic endemicity threshold; the proportion of Rhode Island at risk increased from 6% in 1998 (6 cases) to ≈9% in 2004 (48 cases). The large increase in area at risk for babesiosis that occurred in 2002 (51%) did not unduly increase the number of cases during that year. Several years of abundant nymphal ticks may be needed before *Babesia* *microti* parasites are introduced and established in previously parasite-free mouse populations. Nevertheless, according to 2000 census figures, an average of ≈13% of Rhode Island’s population resides in areas where they could acquire human babesiosis.

Occasionally, babesiosis cases were recorded from northern Rhode Island, where tick abundance is below the proposed transmission risk threshold. These cases are likely to have resulted from travel to areas with dense tick populations or receipt of transfusion (*4*) from donors who lived in *Babesia*-enzootic areas. These cases cannot be accounted for in our research, which is based on the assumption that disease was acquired peridomestically.

A proportion of the increase in human babesiosis incidence during 1998–2004 may be the result of greater awareness of this disease, more correct diagnoses, and more case reporting. To some extent, incidence of babesiosis does seem to be increasing in relation to that of Lyme disease but does not appear to be, as has been suggested, approaching that of Lyme disease in areas of Rhode Island (*8*). Most cases in our sample were in elderly persons (≥60 years of age), in contrast with research by Krause et al., which found that age-to-incidence rate ratio was similar for younger persons (<60 years of age) (*8*). When incidence rates for babesiosis and Lyme disease among patients ≥60 years of age were directly compared, the proportions of patients with Lyme disease who also had babesiosis were 2.4% in 1998, 7.7% in 1999, 18.8% in 2000, 18.8% in 2001, 16.7% in 2002, 12.6% in 2003, and not calculated in 2004 because of different method of recording Lyme disease. This differential is most likely explained by the smaller geographic distribution of *B. microti–*infected ticks compared with *B. burgdorferi–*infected ticks; however, the lower rate of *B. microti* infection in ticks is probably a contributing factor. This lower rate means that more tick bites per person are needed to produce infection.

The years leading up to, and immediately following, the observed reported increase in human babesiosis incidence may have been a period of *B. microti* introduction and enzootic establishment requiring a higher tick risk threshold, which rapidly decreased after establishment. In the absence of statewide reduction of ticks, local expansions of infected rodent populations are likely to continue and further extend the babesiosis risk zone in Rhode Island.
